# Nursing champions to promote evidence-based in-hospital sleep: a qualitative descriptive study

**DOI:** 10.1177/17449871261438394

**Published:** 2026-05-06

**Authors:** Linda Gellerstedt, Åsa Gransjön Craftman, Arja Höglund, Olga Nilsson

**Affiliations:** Assistant Senior Lecturer, Department of Neurobiology, Care Sciences and Society, Karolinska Institutet, Sweden; Senior Lecturer, Department of Neurobiology, Care Sciences and Society, Karolinska Institutet, Sweden; University Nurse, Department of Clinical Neuroscience, Karolinska Institutet, Sweden; Department of Neurology, Karolinska University Hospital, Sweden; University Nurse, Department of Molecular Medicine and Surgery, Karolinska Institutet, Sweden; Department of Vascular Surgery, Karolinska University Hospital, Sweden

**Keywords:** evidence-based nursing, hospitalisation, inpatients, nursing care, sleep, sleep health

## Abstract

**Background:**

Given the significant impact of sleep quality on patient outcomes, integrating evidence-based sleep promotion into routine care practices is essential.

**Aims:**

This study aims to explore sleep nursing champions’ (SNCs) experiences of an evidence-based sleep-promoting project in the context of inpatient hospital care.

**Methods:**

An explorative qualitative descriptive design with an abductive approach, using the Capability, Opportunity and Motivation – Behaviour (COM-B) model as a theoretical framework, was applied. Data were collected through five semi-structured individual interviews and analysed inductively using a reflexive thematic analysis.

**Results:**

Five themes were theoretically interconnected with the determinants of the COM-B model. The findings describe barriers and facilitators in the implementation of evidence-based sleep promotion in hospital care. Motivational and hindering factors included the empowering role of the SNC, organisational structures, time and the interprofessional team, all of which influenced changes to current practice.

**Conclusions:**

Establishing SNCs requires organisational, practical, social and cultural adaptations. When these conditions are met, SNCs can contribute to person-centred, conducive sleep-promoting environments. The results contribute to nursing practice by advancing theoretical understanding of contextual factors shaping sleep-promoting care and by informing future development of evidence-based strategies for improving patients’ sleep in hospital settings.

## Introduction

Patients’ sleep during hospitalisation has been identified in previous research as a significant stressor (Gellerstedt et al., 2014; Morse and Bender, 2019; Stewart and Arora, 2018). The complexities of hospital care often prevent in-patients from obtaining even a few hours of uninterrupted sleep due to necessary interventions such as monitoring, medication administration and other sleep-disturbing factors (Delaney et al., 2018). Consequently, sleep is a crucial yet often neglected aspect of nursing care in acute healthcare settings (Delaney et al., 2018; Gellerstedt et al., 2019; Ritmala-Castren et al., 2022) and patients’ sleep quality is often overlooked (Ritmala-Castren et al., 2017).

## Background

Several reviews indicate that both sleep quantity and quality are insufficient in hospitalised patients (Adams et al., 2024; Burger et al., 2022; Showler et al., 2023). Several reasons for sleep interruption have been described, such as an unfamiliar environment, along with noise, light, pain, anxiety and the performance of various nursing care activities (Little et al., 2012; McLaughlin et al., 2018; Tan et al., 2019; Yilmaz et al., 2012). These often lead to frequent awakenings at night and disturbed sleep (Gellerstedt et al., 2014; Wesselius et al., 2018). A reduced sleep quality can also have negative consequences for the patients, including impaired immune defence (Besedovsky et al., 2012), decreased pain tolerance (Krause et al., 2019), increased risk of confusion and delirium (Knauert et al., 2015), disturbances in insulin regulation (DePietro et al., 2017) and impaired patient recovery (Hillman, 2021; Stewart and Arora, 2018).

Within Virginia Henderson’s Need Theory (Henderson, 1964, 1991), sleep is identified as the fifth of fourteen fundamental human needs, highlighting its essential role in maintaining physiological and psychological health. In a hospital environment where illness, unfamiliar surroundings, noise and interventions frequently disturb patients’ sleep, Henderson’s theory provides a structured way for nurses to assess and address sleep as a core component of holistic care. According to her theory, when patients are unable to meet their own need for rest and sleep, the nurse has a responsibility to support and assist them in achieving adequate restorative sleep. A review emphasises the importance of supporting nursing practice to improve in-patients’ sleep through interventions that enhance nurses’ knowledge, attitudes and confidence in implementing sleep-promoting interventions (Mohedat and Somayaji, 2023). Given the significant impact of sleep quality on patient outcomes, integrating sleep promotion into routine care practices is essential (Beswick et al., 2023; Burger et al., 2022). Moreover, unit norms for sleep promotion can be a significant predictor of the quality of care (Kim et al., 2018). Despite the evidence and knowledge, there remains a noteworthy gap in both awareness regarding in-patients’ sleep and knowledge of sleep-promoting actions among healthcare professionals (Delaney et al., 2018; Gellerstedt et al., 2019).

It is emphasised that forthcoming research of hospitalised patients sleep should direct attention to developing and implementing interventions that bring improvement, and more rigorous research on structural changes in hospital wards is needed (Bellon et al., 2021). Furthermore, nurses can play a crucial role as facilitators in problem solving, enhancing their impact in healthcare and nursing activities. Accordingly, the behaviour change wheel (BCW) including the model Capability, Opportunity and Motivation – Behaviour (COM-B) (Michie et al., 2011) recognises that the target behaviour can arise from combinations of any of the components of the behaviour system. Capability involves an individual’s physical and psychological abilities, including necessary knowledge. Opportunity covers social and physical conditions that facilitate or hinder behaviour, such as resources and norms. Motivation relates to decision-making, planning and incentives for behavioural change (Michie et al., 2011). COM-B can be utilised to explain the conditions that influence behaviours and to identify factors that contribute to behavioural change. Additionally, the model can be employed to describe potential causes of behaviours and how these are managed.

Introducing non-pharmacological sleep interventions is recommended due to its effectiveness, minimal risk and low cost (Jun et al., 2021). Research is needed to evaluate how to optimise the implementation of evidence-based interventions into routine care. This can be done by using a model of care that addresses potential barriers such as contextual factors, differences in ward environments and patient preferences (Beswick et al., 2023). Consequently, nurses need to translate and implement research to guide their clinical practice. Kerr and Rainey (2021) addressed current challenges to implementing evidence-based practice in nursing, including difficulties nurses face in applying evidence to daily practice and the lack of time away from clinical duties to stay updated with research. In the current study, an evidence-based project comprising a sleep-promoting care bundle was implemented in inpatient hospital care. The care bundle consisted of three parts: a clinical practice guideline, e-learning course on sleep for healthcare professionals and implementation of sleep nursing champions (SNCs). Few studies have explored the process of integrating such evidence-based practice into nursing practice, this study therefor aims to address this gap by applying a theoretical framework of behaviour change.

### Aim

To explore SNCs’ experiences of an evidence-based sleep-promoting project in the context of inpatient hospital care.

### Design

An explorative qualitative descriptive design (Sandelowski, 2000) was applied. Interviews with SNCs were conducted through semi-structured individual interviews and analysed with an abductive approach (Graneheim et al., 2017). The study was theoretically underpinned by the COM-B model (Michie et al., 2011) and reported in accordance with COREQ (Tong et al., 2007).

## Method

### Setting

The overarching aim with the project was to position sleep as a fundamental aspect of nursing care, strengthen healthcare professionals’ knowledge of sleep physiology and management and provide practical tools to support sleep-promoting practices in hospital settings. The development and implementation of the project followed the framework, integrated Promoting Action on Research Implementation in Health Services (i-PARiHS) (Harvey and Kitson, 2016), which emphasises implementation strategies to the specific innovation. The intervention was deployed across eight units within the heart and vascular speciality, encompassing neurological, neurosurgical, thoracic, cardiac and vascular surgery departments and the project consisted of three parts. *A web-based educational course*, created in collaboration with an e-learning design agency specialised in pedagogical solutions, consisting of four modules. The modules covered both foundational concepts of sleep physiology and advanced content on sleep disturbances during hospitalisation, supplemented by interactive cases and quizzes to enhance engagement. *An evidence-based clinical guideline* with integrated theoretical principles and practical recommendations for pharmacological and non-pharmacological management of sleep disturbances. The guideline was developed within the project team in partnership with a clinical pharmacist. The content was organised according to the patient’s care trajectory, with sections such as ‘Upon Admission’ and ‘During Ward Rounds’ (Nilsson et al., 2026). *An implementation of Sleep Nursing Champions (SNCs)* with the overarching goal to position sleep as a fundamental aspect of nursing care, strengthen healthcare professionals’ knowledge of sleep physiology and management and provide practical tools to support sleep-promoting practices in hospital settings. The development and implementation process of SNCs was informed by the i-PARiHS framework, which emphasises strategies for translating evidence into clinical practice. The SNCs were recruited from intervention units based on voluntary interest and trained through webinars and in-person sessions. They received scientific resources and a role description outlining responsibilities such as peer education, advocacy for sleep in daily routines and facilitating workflow adjustments to optimise patient rest. Although no dedicated time was allocated for this role, SNCs integrated these activities into their regular duties and remained accessible to colleagues during shifts. The present study focusing on the third part of the intervention.

### Data collection

Through convenience sampling (Polit and Beck, 2020), the SNCs were contacted and invited to participate by the second author via email, which included written information about the study and an informed consent form. Inclusion criteria were Registered Nurses (RNs) who had held the role of SNC for 6 months or more. In total, nine out of a total of eleven implemented SNCs met the criteria. All participants were given the opportunity to choose a location for interview, either in person at a local site or through a digital platform (Zoom). Four out of nine prospective participants declined to take part due to excessive professional commitments. Interested participants contacted the second author to arrange a time and place for the interview. In total, five participants were included in the study. The interviews were based on a semi-structured interview guide (Table 1) developed within the research group. Prior to data collection, the interview guide was tested for face and content validity (Polit and Beck, 2020) by three specialist RNs with extensive working experience in acute care hospitals. They were invited to comment on the content and any ambiguities arising from the phrasing of the questions. No revisions were needed after review.

**Table 1.** Interview guide.

Key questions	Domains in the COM-B model
How did you become interested in sleep?	Motivation
The knowledge that you have about sleep, how did you acquire it?	Capability
What does it mean to be a SNC?	Opportunity
Do you have time for your assignment? If not, how have you handled it?	Opportunity
How do you as SNC work with sleep in the department/ward?	Capability/Opportunity
How do you work to disseminate knowledge about sleep to your colleagues?	Opportunity/Motivation
Have you had time to follow up and evaluate – if so, how did you do it and how did it go?	Opportunity/Motivation
How do you work with sleep promotion? Give examples of sleep-promoting interventions, when and how you use these?	Capability/Opportunity
Do you feel that you work in a different way after SNC was implemented – How has the introduction of SNC affected your way of working regarding patients’ sleep?	Opportunity/Motivation
How do you feel that the introduction of SNC has been received by colleagues?	Capability/Motivation
How do you feel that you, as SNC, are supported in your function?	Opportunity
What do you consider to be facilitators for succeeding in the assignment?	Capability/Motivation
What do you consider to be obstacles for succeeding in the assignment?	Capability/Motivation
What barriers or setbacks have you encountered in the assignment? How have you handled this?	Capability/Motivation
In what way do you think that patients’ sleep is affected by the fact that SNC has been introduced in your department?	Motivation/Opportunity
Is there anything else you would like to add or talk about regarding your role as SNC that we have not covered?	

Before each interview began, verbal consent was obtained, and the participants signed the informed consent form. Each interview started with questions regarding demographics and was followed by the question, ‘Could you please tell me how you became interested in sleep?’ The interview guide was followed throughout all interviews, but not in chronological order; rather, all questions were asked to enhance the flow of the conversation. To obtain deeper and more extensive descriptions, probing and follow-up questions were used, such as ‘Could you describe further and/or provide an example?’ All participants chose to be interviewed via Zoom, and the interviews lasted between 41 and 97 minutes (mean 68 minutes). All interviews were conducted by the second author between February and April 2024 and were audio-recorded with the participants’ approval.

### Analysis

To gain a deep understanding of experiences regarding SNCs’ assignment and their work with sleep promotion, and with COM-B as a theoretical framework, an abductive analysis (Graneheim et al., 2017) was applied. Firstly, an inductive approach with a reflexive thematic analysis (Braun and Clarke, 2006, 2019) comprising the described six phases was applied. In the sixth phase of the thematic analysis, the analysis was shifted to a deductive approach to map and interconnect generated themes on to the COM-B model.

**Figure 1.**
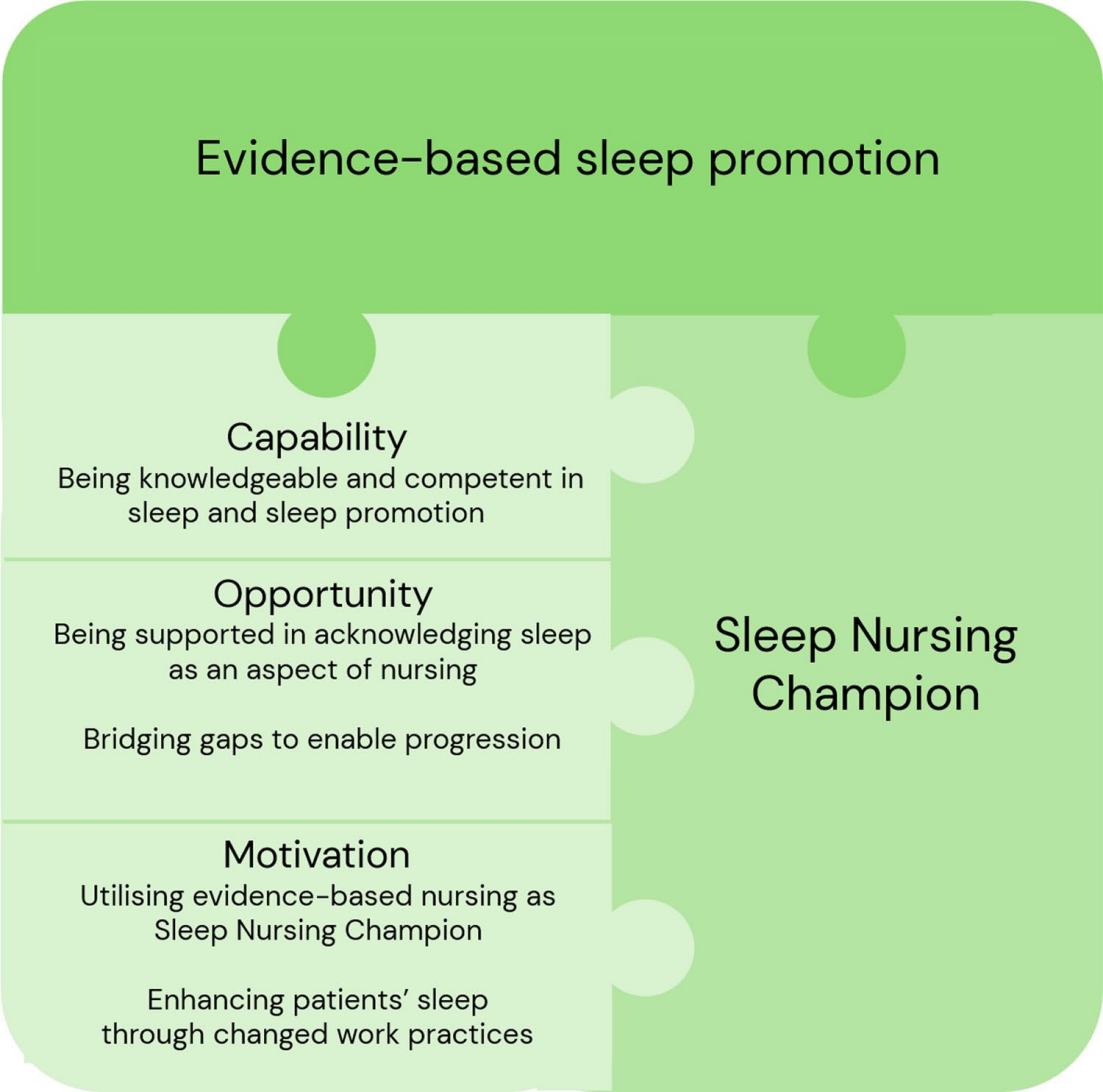
The five formulated themes interconnected with the determinants of capability, opportunity and motivation (COM-B model).

The analysis was undertaken by the first and second authors and was performed manually (i.e. no qualitative software programme was used). Initially, in Phase 1 (Familiarisation with data), the interviews were transcribed verbatim, and thereafter both authors individually, and repetitively read the transcripts for in-depth familiarisation with the data. In Phase 2 (Generating initial codes), in line with the aim, segments of data were identified, and the initial coding was first independently generated. Thereafter, the codes and segments of data were compared, discussed and decided on and transferred into a Microsoft Excel file, serving as a coding sheet. During Phase 3 (Searching for themes), the codes were cut into pieces of paper and by hand, the codes were grouped to inherent themes, and these assembled themes formed a thematic map. During Phase 4 (Reviewing themes), the thematic map was discussed and revised by all four authors resulting in five themes with underlying sub-themes. In Phase 5 (Defining and naming themes), coherent sub-themes were collapsed under the five themes, and these themes were re-formulated. In Phase 6 (Producing the report), the analysis shifted into a deductive approach aiming to interlink elements in the formulated themes onto the theoretical determinants’ capability, opportunity and motivation based on the COM-B model (Figure 1).

### Rigour and reflexivity

To strengthen qualitative rigor and to facilitate the analysis process, the analysis was performed in line with the six-phase process according to Braun and Clarke (2021). Trustworthiness in qualitative research is described through four domains: confirmability, credibility, dependability and transferability (Lincoln and Guba, 1985). Credibility was enhanced due to the authors’ proficiency in qualitative interviewing and analysis. Dependability was strengthened by the author conducting the interviews, had no pre-conception regarding the research area sleep, nor the concept of SNC. Furthermore, thorough descriptions of the research process enhance the credibility of the findings, including the analysis and how the theoretical framework was incorporated. Regarding confirmability, quotes were used to provide a close and rich understanding of the themes’ content and to demonstrate how the findings were clearly derived from the transcribed data. All authors reviewed and discussed the analysis in a reflexive manner, engaging in an iterative analytic process, moving repeatedly through the data while critically examining and refining emerging meanings and interpretations. Extracts and codes have been discussed and verified after the themes were formulated. To enhance transferability, both the context and the intervention have been thoroughly described. Thus, the findings from this study are not intended to be generalised; rather, it is for the reader to assess if and how the findings are transferable to other similar contexts.

### Ethical considerations

The study was approved by the Swedish Ethical Review Authority (Dnr:2023-04670-01) and performed in accordance with the Helsinki Declaration (World Medical Association, 2025). All participants provided written informed consent, and the findings are presented in a way that ensures no participant can be identified.

## Findings

The five themes were interconnected with the determinants of capability, opportunity and motivation in the COMB model. Although presented separately, the themes were closely interrelated. Across the themes, the SNCs described how their capability, opportunity and motivation shaped their work with sleep and sleep promotion. Theme 1, highlighting the SNCs’ growing competence, linked directly to Theme 2, where collegial support and the SNC network reinforced their confidence and ability to prioritise sleep. Theme 3 demonstrated how capability was constrained by limited organisational opportunity, such as challenges in coordinating across shifts and engaging physicians. Motivation, reflected in Themes 4 and 5, both stemmed from and reinforced the preceding themes. Engaging with evidence-based nursing strengthened their commitment and helped them navigate organisational barriers (Theme 3), whereas observing improvements in patients’ sleep enhanced their sense of capability (Theme 1) and underscored the importance of support (Theme 2). Together, the themes illustrate how capability, opportunity and motivation interacted dynamically as the SNCs advanced their work in sleep promotion.

In total, five participants were included in the study and characteristics of the participants are presented in Table 2.

**Table 2.** Characteristics of the participants.

Demographic variables	Total *n* = 5
Age	Mean 49 years (range 34–57)
Educational level	
Registered nurse (RN)	4
Specialist nurse	1
Professional experience in years as a nurse	Mean 14.1 years (range 4,5–26)
Working schedule	
Day and evening shift	1
Three shifts (day, evening and night shift)	2
Night shifts only	2
Clinical settings/wards	
Neurology	2
Thoracic surgery	1
Cardiology	1
Vascular surgery	1

### Theoretical interconnection of themes on the COM-B model

## Capability

### Theme 1: Being knowledgeable and competent in sleep and sleep promotion

Sleep was described as a fundamental need, and the SNCs expressed it as noteworthy that the area had been given such modest priority in the past. Nurses who held the assignment as SNC described their ability and knowledge in working with sleep promotion when caring for patients. They emphasised the importance of actively listening to the patients, showing interest in and assessing patients’ sleep to gain a comprehensive understanding. They expressed how their competence in the field naturally led them to ask patients about their sleep habits at home, the strategies they use, and how they manage sleep difficulties.

Truly taking an interest in their sleep, asking questions, and to follow up. In this way, we show that sleep is important, and it becomes easier for them to open up. (4)

The SNCs stressed the importance of communication and providing information about what was planned, as this creates a sense of security for patients, which in turn becomes a sleep-promoting intervention. Creating a calm and predictable care environment in a situation that may be, for many, both unfamiliar and frightening was described as essential. Additionally, discussing what is planned for the night and involving the patients in their own plan was described as an effective sleep intervention.

Creating a calm room, providing information about what will happen during the night and in the morning. This makes them feel safer and able to sleep. (5)

The participants shared that their understanding of how patients’ sleep is affected indicated that all staff, regardless of shift, need to be more attentive and work together to promote the patient’s sleep. Examples of measures mentioned included actively working with mobilisation and activation during the day to maintain a stable circadian rhythm, as well as using daylight. There were also suggestions to change environmental factors, such as lighting, and to ensure more natural daylight on their wards. One SNC described this as:

To work with sleep-promotion during the day, pulling up the blinds and ensuring they get dayligh. (3)

Additionally, the importance of involving and engaging all categories of healthcare professionals was voiced. This was expressed as necessary both because sleep is influenced by events throughout the day and because patients’ sleep is a shared responsibility that should be upheld by everyone. Having extensive nursing experience was seen as an empowering factor that provided confidence in both the role and its associated responsibilities:

I am knowledgeable, I know more now. I perceive that colleagues’ sense that I am someone they can ask and turn to. Now, I can promote the area. (1)

## Opportunity

### Theme 2: Being supported in acknowledging sleep as an aspect of nursing

The SNCs described the initial implementation phase of the new role as vulnerable, requiring a range of support. The importance of being able to rely on other SNCs and the feeling of being a part of a network was also voiced. They addressed how they were inspired by their fellow SNCs and had a desire for an extended collaboration between departments that have this role implemented. It was essential to feel supported by the project group that developed and initiated the project, and the shared digital workspace was one way to stay connected with the project team and the other SNCs. This collaborative process enabled the SNCs to share good examples, reflect together and motivate one another, thereby strengthening collective learning and supporting the development of professional competence and nursing practice. One SNC said,

They (the project group) do not dictate what we should do, but instead offer suggestions, ideas, and encouragement. (4)

Experiencing support from their direct manager was highlighted as a key factor, with SNCs feeling that their manager genuinely believed in the project. One SNC described how, as nurses are problem-solvers so why they should not be able to solve this issue around patient sleep? Several SNCs pointed out that there is scientific evidence supporting sleep-promoting interventions, and they felt the project was taken seriously because it was based on evidence.

### Theme 3: Bridging gaps to enable progression

One obstacle in achieving success within the project was described by the SNC as difficulties in coordinating with colleagues across different shifts, especially when some were working night shifts only. SNCs wanted sleep to be addressed during the medical rounds in the same way that pain and nutrition are. However, it was challenging to communicate the importance of patients’ sleep to the medical doctors on the ward, as they were neither familiar with sleep-related issues nor aware of the significance of sleep for patients’ recovery. The SNCs nevertheless expressed a desire to include them as essential partners and stakeholders in the project. The participants advocated for appointing nurses as SNCs in all wards across the hospital, as the project group and the associated network provided an important shared platform for exchanging knowledge and collaboratively developing nursing practice related to sleep. One SNC reflected on the future by stating:

Every care unit in the entire hospital should have a dedicated sleep nurse, and ideally, there should be two – one for the day shift and one for the night shift. (5)

Several SNCs described working on developing the area of sleep and their professional role during periods of unallocated time in their schedules. They often relied on what they referred to as ‘*stolen time*’, meaning that tasks were completed outside the formally allocated schedule by using breaks, personal time or by overlapping responsibilities. It was also common for the nurses to engage with the theoretical component of the project during their leisure time, for example by completing assignments at home or reviewing materials on their days off:

Dedicated time is not scheduled, but sometimes I have several days off in a row, and occasionally the nights on the ward have calmer periods, so I can work on it then. (5)

All SNCs emphasised the necessity for more dedicated time to enable progress in the project.

## Motivation

### Theme 4: Utilising evidence-based nursing as SNC

The participants described how the role as SNC had given them both the opportunity to be part of a new way of addressing patients’ sleep and a chance to influence how sleep nursing care is provided. They perceived that their commitment to the area was strong and had deepened, and one SNC described the involvement as:

Being part of bringing attention to an area that was previously a low priority that actually could contribute to improved care and nursing. (4)

They shared how their approach to the assignment, emphasising facilitating factors, related both to their personal attributes and to external conditions that supported their efforts. Some described how the role gave them a sense of authority when implementing various measures and interventions, and how they could serve as a sounding board for nursing colleagues.

In the role as a SNC, I feel that I have power and can argue that the patient's sleep must be prioritized, and I dare to take up space. (5)

Another motivational factor was possessing a personal drive and passion for sleep care, along with an openness to and interest in driving change.

### Theme 5: Enhancing patients’ sleep through changed work practices

Some of the SNCs expressed that they sympathised with patients who were unable to sleep, but also how this, in a way, strengthened their devotion to place sleep high on their agenda of nursing. During the interviews, it was voiced that the implementation of SNC had made a noteworthy impact on both individual patients’ sleep and the daily work practices of sleep on the wards. By reflecting together as a team and engaging in discussions with colleagues, SNCs described how the area of sleep had gained more focus and magnitude. Furthermore, they felt encouraged when they noticed that patients’ sleep was more frequently considered and addressed during admissions, rounds and in nursing documentation. SNCs stated that shared discussions, combined with increased knowledge, had led to changes in practices related to sleep, such as fewer blood samples being taken early in the morning, and the same applied in monitoring vital signs, which now were adjusted whenever possible to enhance patients’ sleep.

Now since SNC is implemented, we postpone early blood tests and checks of vital signs for patients who really need their sleep. (5)

The SNCs described how they were advocating for and encouraging staff to complete the digital sleep course. Several SNCs mentioned it as a goal within their role for all nurses on the ward to complete the course. In some wards, the sleep course had been made mandatory for newly employed nursing staff. One SNC explained how the introduction of the responsibility for sleep had led to an increased awareness of physical factors disturbing sleep. Their prior experience, along with new and expanded knowledge, had resulted in environmental factors being given more consideration, with an additional proactive approach being taken. Moreover, SNCs reported an extended understanding of patients’ sleep among all nursing staff, not just those working night shifts only.

We have become more aware of things like lighting, and we remind each other to keep our voices down to avoid disturbing the patients. It’s a new way of thinking, with a completely different focus. We’ve identified a problem area, are working to develop it, and yes, now we can see changes. (4)

One area that brought motivation was descriptions of how they plan the night shifts differently than before. Nursing and medical interventions that could disturb patients’ sleep were increasingly coordinated and planned. Some SNCs mentioned that organised daytime rest had been implemented to allow patients to rest during daytime. Other motivational changes since the introduction of SNC was that in some wards, general prescriptions for sleep medication had been changed from Z-drugs (a group of prescription medications used to treat sleep problems) to melatonin (a synthetic version of the natural sleep hormone used to help regulate the body’s sleep–wake cycle). The administration of sleep medications had generally decreased and was no longer given routinely. One SNC described this change as:

Now, we first have a conversation before giving a tablet. We have changed, we think more carefully first, and we highlight other sleep enhancement as alternatives. (3)

## Discussion

The present study aimed to describe the experiences of nurses holding the SNC role to implement evidence-based sleep promotion in in-patient care. The SNCs who participated identified feasibility factors that affected change to current practice. These included the empowering role as SNC, the organisation, time and the interprofessional team. Consequently, nurses need to translate and implement research to guide their clinical practice. Kerr and Rainey (2021) addressed current challenges to implementing evidence-based practice in nursing, such as difficulties nurses face in applying evidence to daily practice and the lack of time away from clinical duties to stay updated with research.

Nurses play a crucial role within the interdisciplinary team, representing the largest group of healthcare professionals, providing round-the-clock care and spending significant time in bedside patient interaction (Hommel et al., 2020). There are various approaches to take in developing and implementing sleep promotion interventions where nurses’ competence is in focus. The SNCs in this study recognised the benefits of the project group and other SNCs for support and discussions which is consistent with former studies. Busca et al. (2021) and Dijkstra et al. (2006) identified the necessity to consider multiple factors, including nursing workload, education, funding, professional trust and mutual respect across various domains such as interprofessional, interpersonal and organisational when developing a customised intervention. SNC participants identified barriers related to limited time, communicating sleep within the interprofessional team and changing suggested sleep medication of the general prescription to non-pharmacological interventions which have proven effective in enhancing subjective sleep quality and reducing the incidence and duration of delirium (Kang et al., 2023). The project’s foundation in research empowered the SNCs and provide the project a legitimacy within the organisation.

According to our findings, the SNCs own interest in, and knowledge of, the effects of sleep on patients’ health and recovery were clearly demonstrated and aligned with Henderson’s (1991) Need Theory regarding fundamental nursing needs. Furthermore, the findings illustrate how clinically practising nurses can apply and operationalise a nursing theory within a clinical context. Their interest and knowledge about patients’ sleep, combined with the perceived low priority given to the area, served as strong motivation to elevate its importance as an SNC. Sleep was addressed in daily work practices during admissions, rounds, handovers and in nursing documentation. Sleep was also included in the patients’ care plans, which is also suggested by Ritmala-Castren et al. (2022) to acknowledge sleep as an important aspect from both patient and nursing perspective.

The SNCs collaborated within the interprofessional team to identify and address nocturnal disruptions caused by modifiable standard clinical activities to provide patient-centred sleep promotion. This finding is in line with that by Holleck et al. (2023), suggesting that vital sign monitoring and medication administration should be moved outside of normal sleeping hours to minimise nocturnal disruptions.

To progress this evidence base, research is needed to evaluate how the implementation of evidence-based interventions can be implemented into routine care, using a model of care that addresses potential barriers such as contextual factors, differences in ward environments and patient preferences (Beswick et al., 2023). A systematic review and network meta-analysis of 31 articles (Huang et al., 2023) of improving the sleep quality and overall well-being of critically ill patients suggests that nurses should implement tailored interventions. They also recommend assessing the impact of contextual factors and refining interventions based on patient feedback and outcomes. Similar conclusions are drawn by Bellon et al. (2021) who add the need for research on structural changes in hospital wards. The current project gave perquisites for the SNC to place in-patients sleep on the agenda of nursing. However, it requires both personal and organisational changes.

### Strengths and limitations

Our study has both strengths and limitations. A strength of the study lies in the use of a theoretical framework. By interconnecting the formulated themes with the COM-B model, the findings are presented in relation to key components of behavioural change. The themes further demonstrate that meaningful behavioural change in sleep nursing practice occurred when all three elements of the COM-B model were present and interacting. A potential limitation of the study is the gender composition of the participants. The participating SNCs constituted a homogeneous group in terms of gender but reflected the current gender distribution within the nursing profession in Sweden. Furthermore, the study was conducted at a hospital in a metropolitan area and within a limited context of medical specialties. In terms of data saturation, the study’s aim dictates the optimal amount of data to be collected, and the exact number of participants or interviews required in a qualitative study should not be predetermined (Hennink and Kaiser, 2022). In this pilot project, the number of implemented SNCs who fulfilled inclusion criteria was limited, and no further participants could be included. Our results are therefore limited by the restricted sample and may be interpreted with caution regarding transferability. To ensure trustworthiness, a predesigned and validated interview guide was utilised. Due to credibility, another strength of the study was that the interviewer, ÅGC had neither preunderstanding of the area of sleep or the project nor any previous contact with the participants, which may enhance the credibility of our findings.

## Implications

This study highlights key contextual determinants influencing the implementation of evidence‑based sleep promotion in hospital care. Strengthening organisational support, interprofessional collaboration and the structural conditions that enable SNCs may promote sustainable practice change and foster more sleep-conducive, person-centred inpatient care environments.

The findings underscore the need to strengthen nursing education on sleep, particularly in sleep assessment, sleep physiology and evidence-informed non-pharmacological interventions. Educational programmes should include structured training that helps nurses understand contextual determinants of sleep-promoting care and develop the competence needed to apply such practices in complex inpatient settings. Improved educational preparation may better equip nurses and future SNCs.

Effective sleep-promoting nursing practice relies on the organisational, social and practical conditions that enable SNCs to act as catalysts for behavioural change. Their experiences emphasise the need for clear role definitions, supportive teamwork and sufficient time and resources to integrate evidence-based sleep strategies into routine inpatient care. Strengthening these contextual factors may enhance nurses’ capability and opportunity to implement sleep-conducive interventions and ultimately contribute to improved patient outcomes.

Healthcare systems need to recognise sleep promotion as a core element of high-quality, person-centred inpatient care. Effective integration of SNCs requires organisational structures that support behavioural change, including clear role allocation, protected time and interprofessional collaboration. Policies that prioritise sleep-conducive environments, emphasise non-pharmacological approaches and strengthen implementation capacity may enhance the consistency of evidence-based sleep care and improve patient outcomes.

Future research should examine how SNCs influence patient outcomes and care processes across varied hospital settings. Studies of organisational readiness, implementation mechanisms and long-term sustainability are needed to understand how evidence-based sleep promotion can be embedded in routine practice. Comparative and longitudinal designs may also clarify which contextual determinants most strongly support successful behavioural change and the wider adoption of sleep-promoting interventions.

## Conclusions

Implementing an evidence-based but scarcely prioritised area into nursing clinical practice is a pioneering task. The establishment of the SNC role in a hospital context requires organisational, practical, social and cultural adaptations. If these are in place, SNCs have the potential to attain person-centred, conducive sleep-promoting environments. In this context, interprofessional collaboration and allocated time for the project are key elements to enhance sleep-promoting activities in nursing practice.

**Key points for policy, practice and/or research**

- Sleep is a fundamental yet historically overlooked aspect of nursing care; the Sleep Nursing Champion (SNC) role strengthened nurses’ capability in sleep promotion.
- Support from colleagues, managers and the SNC network enabled shared learning and increased opportunities to prioritise sleep.
- Limited interprofessional engagement and coordination across shifts restricted implementation, highlighting the need for clearer structures.
- Motivation was strengthened through evidence-based practice and visible improvements in patients’ sleep.
- A systematic organisational approach with dedicated time could enhance sustainable improvements in sleep-related care.
